# Supplementary material for: JunB Controls Intestinal Effector Programs in Regulatory T Cells
Source: Front Immunol. 2020 Mar 31;11:444. doi: 10.3389/fimmu.2020.00444 (PMC7137613; doi:10.3389/fimmu.2020.00444)

Supplementary Figure Legends

Supplementary Fig. 1. Expression of JunB in Treg subsets and correlation between JunB-dependent weight loss and proinflammatory cytokine production.

(A) Expression of JunB in cTreg and eTreg cells, pre-gated as $TCR\beta^{+}CD4^{+}Foxp3^{+}$ cells. Data are aggregated from 8 mice from 2 independent experiments. Significance determined by two-way ANOVA with Tukey's HSD. **(B)** Association between colonic proinflammatory cytokine production and decreased body weight. Shown are proportions of cytokine-producing cells amongst $TCR\beta^{+}CD4^{+}$ cells in the colon. Data are aggregated from at least 8 mice per group from 4 independent experiments. All summary data represent mean with a bootstrapped 95% confidence interval. **p < 0.01.

Supplementary Fig. 2. Altered Treg and cytokine responses following Treg-specific JunB deletion.

(A) Total cell count from the indicated organs in HET and KO mice. Data are aggregated from at least 6 mice per group from 8 independent experiments. Statistical significance is based on a significant ANOVA interaction term followed by planned comparisons within each organ using Welch's *t*-test with Holm-Bonferroni correction. **(B)** Frequency of $KLRG1^{+}$ cells among $TCR\beta^{+}CD4^{+}Foxp3^{+}$ cells. Data are aggregated from at least 5 mice per group from 3 independent experiments. Statistical significance determined by Mann-Whitney U-test with Holm-Bonferroni correction. **(C)** Comparison of relative CD44 expression on cTreg and eTreg cells from HET and KO mice. Data are aggregated from at least 8 mice per group from 4 independent experiments. **(D)** Correlation of the indicated cytokines with the abundance of $ST2^{+}$ Treg cells. Spearman's ρ was calculated for pooled values from both genotypes and all organs, and indicated a significant correlation in all cases shown ($p < 1\times10^{-5}$). At least 4 animals per genotype from 3 separate experiments. **(E)** Frequency of $Helios^{+}$ cells among $TCR\beta^{+}CD4^{+}Foxp3^{+}$ cells. Data are aggregated from at least 7 mice per group from 4 independent experiments. Significance determined by Mann-Whitney U-test with Holm-Bonferroni correction. All summary data represent mean with a bootstrapped 95% confidence interval. *p < 0.05; **p < 0.01; ***p < 0.001; ns, not significant.

Supplementary Fig. 3. Loss of JunB in Tregs alters germinal center responses and Treg phenotypes.

(A) Frequency of PD-1^{hi}Bcl-6^{+} or PD-1^{hi}CXCR5^{+} Tfh cells among TCRβ^{+}CD4^{+}Foxp3^{-} cells. Data are aggregated from at least 7 mice per group from 4 independent experiments. **(B)** Frequency of CD38^{-}Bcl-6^{+} GC B cells among B220^{+} B cells. Data are aggregated from at least 7 mice per group from 4 independent experiments. **(C)** Frequency of IgG1^{+} GC B cells (defined as in *(B)*). Data are aggregated from at least 5 mice per group from 2 independent experiments. **(D)** Frequency of CD38^{+}IgG1^{+} memory B cells among B220^{+} cells. Data are aggregated from at least 5 mice per group from 2 independent experiments. Statistical significance determined using Mann-Whitney U-test with Holm-Bonferroni correction. **(E)** Representative contour plots (left) and summary data (right) showing IgA and Igκ binding to DAPI^{+} microbes isolated from feces of Rag1-KO mice, JunB HET, and JunB KO mice. Data are aggregated from 4 Rag1-KO, 8 HET, and 6 KO mice from 2 independent experiments. **(F)** Volcano plot of RNA-seq data comparing PP Treg cells from HET and KO mice. Genes with FDR < 0.05 are shown in blue, with select genes highlighted in orange. *p*-values were capped at $1x10^{-40}$ for visualization. **(G)** Expression level of *Icos* in PP Treg cells from HET and KO mice, as determined by RNA-seq. TPM, transcripts per million. **(H)** Frequency of IL-2-expressing TCRβ^{+}CD4^{+} T cells as determined by flow cytometry. Data are aggregated from at least 6 mice per group from 3 independent experiments. Statistical significance determined by Mann-Whitney U-test with Holm-Bonferroni correction. **(I)** Representative contour plot showing the relationship between CD25 and ICOS expression. **(J)** Relative expression of ICOS on CD25^{+} and CD25^{-} Treg cell subsets. Data are aggregated from at least 9 mice per group from 4 independent experiments. Significance determined by Mann-Whitney U-test with Holm-Bonferroni correction. All summary data represent mean with a bootstrapped 95% confidence interval. *p < 0.05; **p < 0.01.

Supplementary Fig. 4. Inducible deletion of *Junb* recapitulates key phenotypes of JunB-KO Tregs.

(A) Activity of *Foxp3$^{YFP\text{-}cre}$* across hematopoietic lineages, assessed by FACS as the proportion of ZsGreen^{+} cells within the indicated cell type. Data shown are from one experiment. Data represent 3 mice from one experiment. **(B)** Efficiency of tamoxifen-induced Cre activity, assessed by flow cytometry as the proportion of Foxp3^{+} cells that were labeled with ZsGreen 14 days after tamoxifen administration. Data are aggregated from at least 14 mice per group from 7 independent experiments. **(C)** Number of Foxp3^{+}ZsGreen^{+} cells across

organs in iHET and iKO mice. Data are aggregated from at least 15 mice per group from 7 independent experiments. **(D)** Total cell number in the indicated organs of iHET and iKO mice. Experimental replicates are the same as in *(C)*. **(E)** Frequency of Ki-67-expressing cells among conventional or regulatory $CD4^+$ T cells. Data are aggregated from at least 4 mice per group from 2 independent experiments. Statistical significance determined by Mann-Whitney U-test with Holm-Bonferroni correction. **(F)** Frequency of eTreg cells among $Foxp3^+ZsGreen^+$ cells (left) and relative gMFI of CD44 expression on eTregs (right). Statistical signifiance determined by Welch's *t*-test. Data are aggregated from 11 mice per group from 4 independent experiments. **(G)** Frequency of $ROR\gamma t^+$ cells among $Foxp3^+ZsGreen^+$ cells determined by flow cytometry. Data are aggregated from at least 6 mice per group from 4 independent experiments. Statistical significance determined by Mann-Whitney U-test with Holm-Bonferroni correction. **(H)** Frequency of $ST2^+$ cells among $Foxp3^+ZsGreen^+$ cells determined by flow cytometry. Data are aggregated from at least 6 mice per group from 5 independent experiments. **(I)** Frequency of IL-2-expressing cells among $TCR\beta^+CD4^+ZsGreen^-$ cells. Data are aggregated from at least 7 mice per group from 3 independent experiments. Statistical significance determined by Mann-Whitney U-test with Holm-Bonferroni correction. **(J)** Relative gMFI of ICOS on $Foxp3^+ZsGreen^+$ cells. Data are aggregated from at least 13 imce per group from 5 independent experiments. Statistical significance determined by Mann-Whitney U-test with Holm-Bonferroni correction. **(K)** Relative gMFI of PD-1 on $Foxp3^+ZsGreen^+$ cells. Data are aggregated from at least 14 mice per group from 5 separate experiments. Statistical significance determined by Mann-Whitney U-test. **(L)** Transcripts per million (TPM) of select effector genes in PP Tregs as determined by RNA-seq, orange asterisks indicate genes identified as significantly different by DE analysis (FDR < 0.05). All summary data represent mean with a bootstrapped 95% confidence interval. *p < 0.05; **p < 0.01; ***p < 0.001; ns, not significant.

Supplementary Fig. 5. Regulation of gene expression in $CD25^-$ PP Tregs and colonic Tregs by JunB.

(A) Frequency of Tregs determined by FACS at day 7 post-tamoxifen administration. Data are aggregated from 4 mice per group from 2 independent experiments. **(B)** Expression of JunB in $TCR\beta^+CD4^+Foxp3^+ZsGreen^+$ Treg cells at day 7 post-tamoxifen, determined by flow cytometry. Data show 2 mice per group from 1 experiment. **(C)** GSEA analysis of RNA-seq data comparing $CD25^-$ PP Tregs from iHET and iKO mice.

Pathways in blue are underrepresented in iKO, whereas those in orange are overrepresented. All pathways that reached statistical significance are shown (FDR < 0.25, *p*-value < 0.05). **(D)** GSEA analysis of RNA-seq data comparing Tfr cells and Treg cells. Pathways in orange are associated with Tfr cells, whereas those in blue are associated with non-Tfr Tregs. All pathways that reached statistical significance are shown (FDR < 0.25, *p*-value < 0.05). **(E)** Volcano plot showing differential expression in colonic Tregs from iHET vs iKO mice at day 7 post-tamoxifen. Genes with significant DE (FDR < 0.05) are highlighted in orange, with select genes labeled. **(F)** *t*-SNE plot showing clusters of cells from scRNA-seq used to assess the distribution of JunB-dependent gene expression across Treg subsets. [Interferon-stimulated genes (ISG); lymphoid-tissue (LT)] **(G)** Dot plot showing the expression of canonical markers used to assign cell identities to clusters from scRNA-seq as in *(F)*. All summary data represent mean with a bootstrapped 95% confidence interval.

Supplementary Figure 1

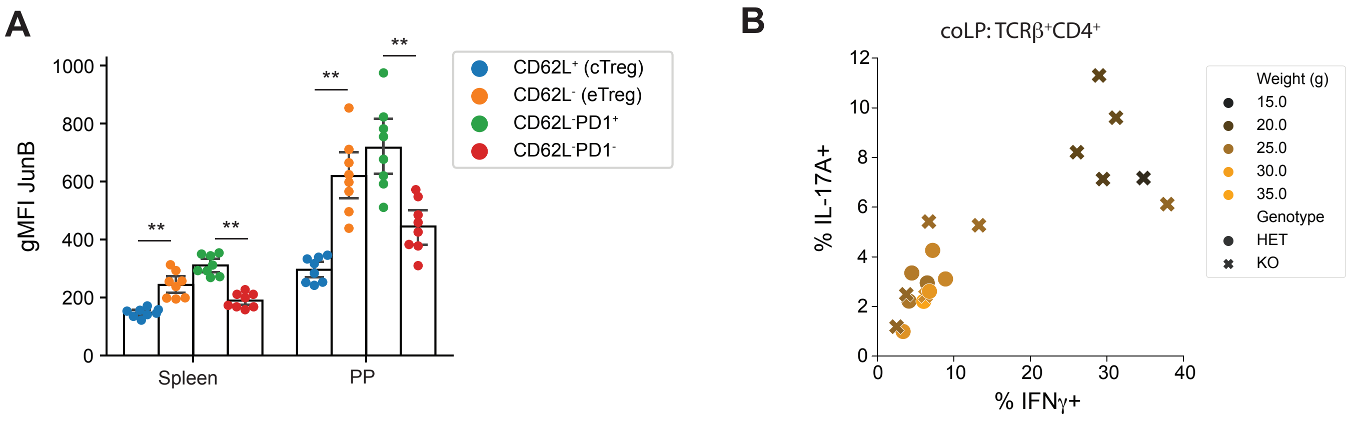

Supplementary Figure 2

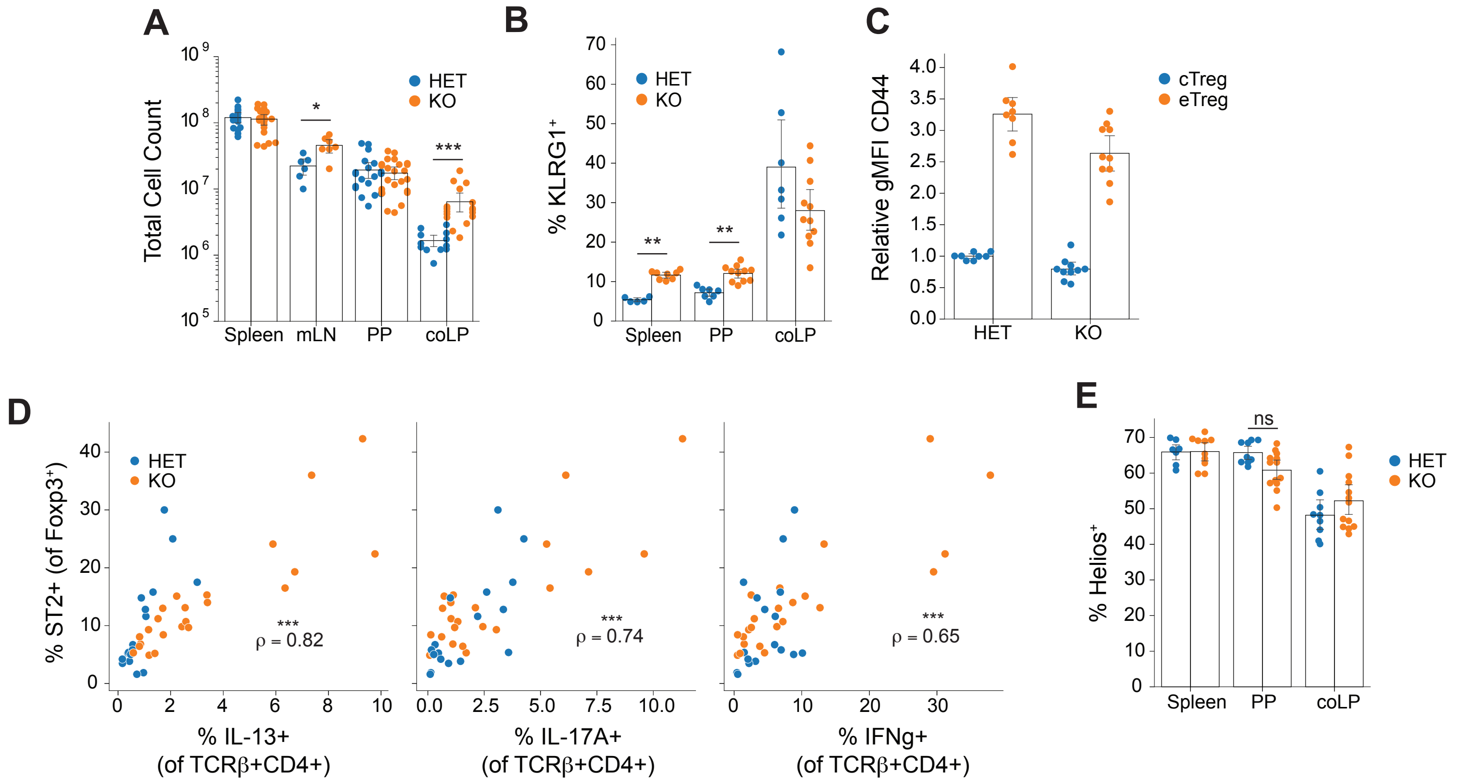

Supplementary Figure 3

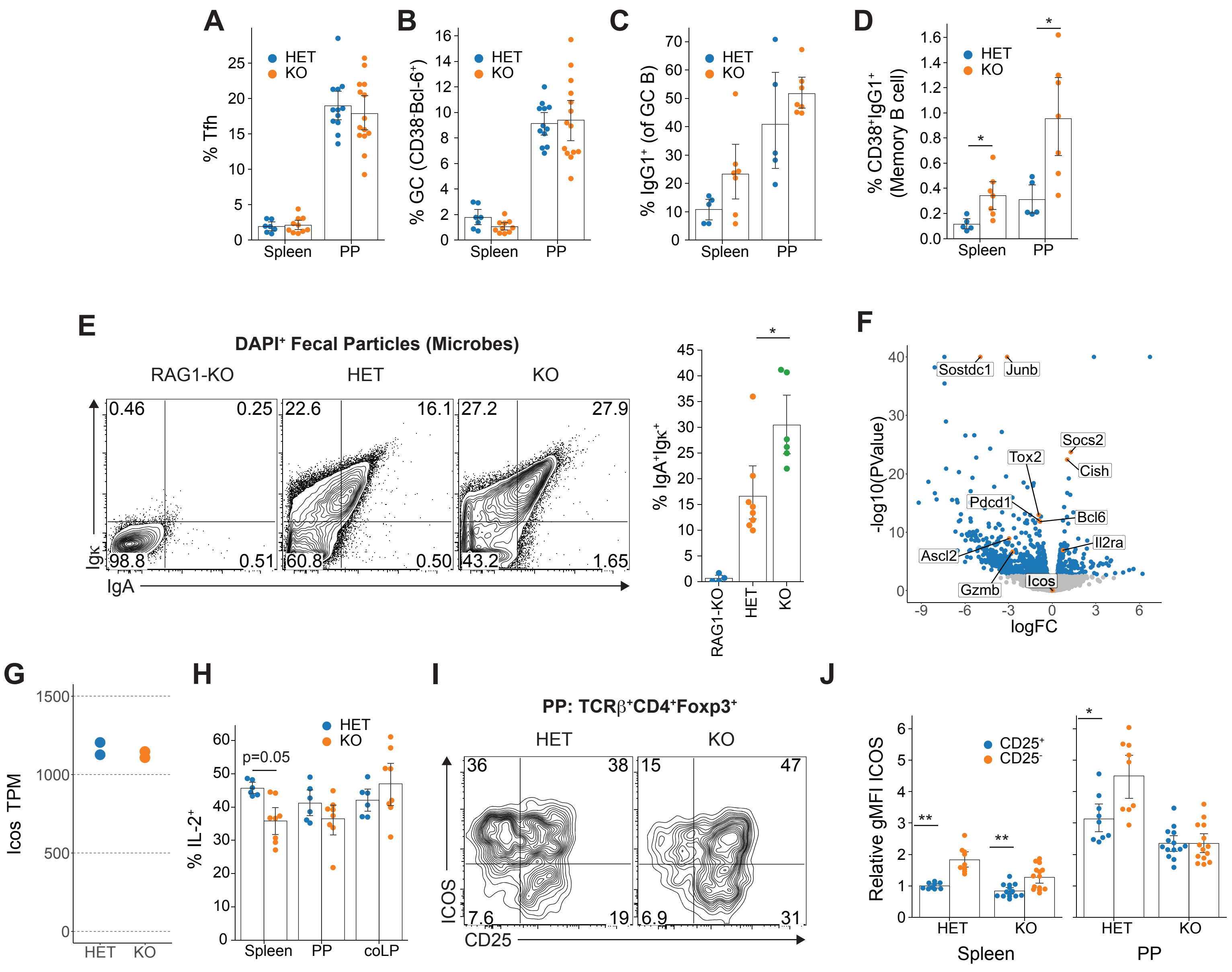

Supplementary Figure 4

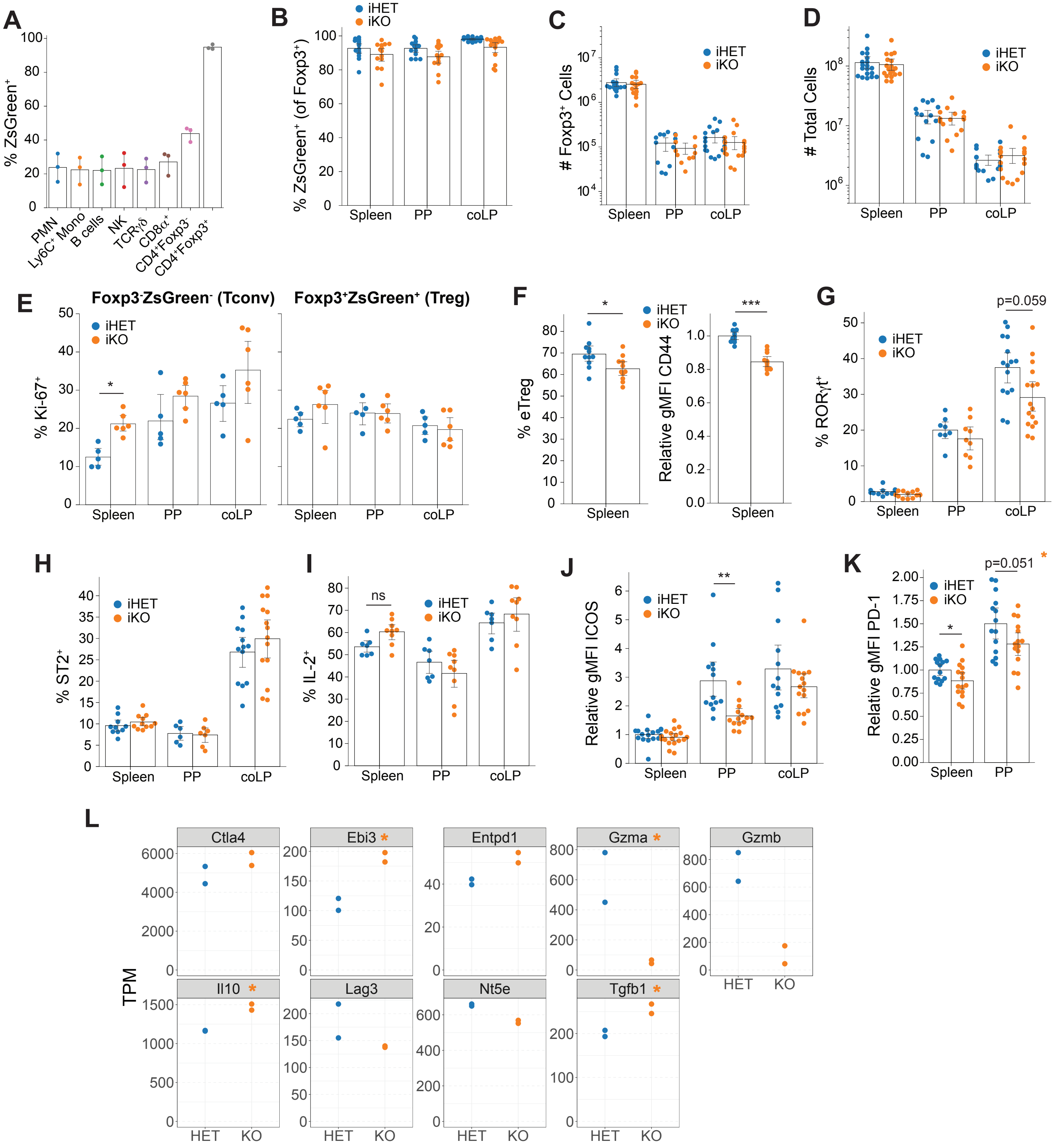

Supplementary Figure 5

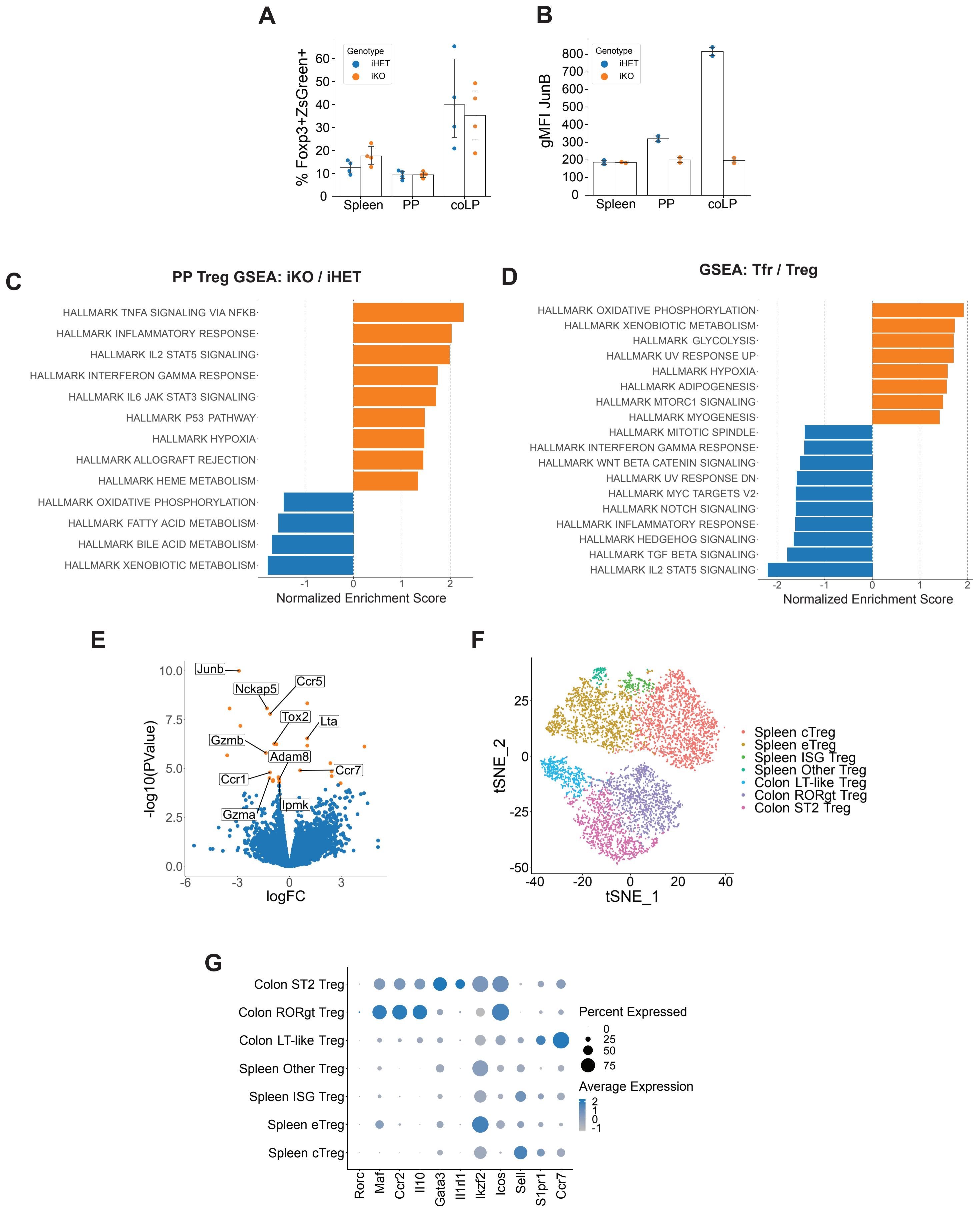

Supplement: Supplementary file 1 [file Data_Sheet_1.PDF]
